# Glycaemic control in people with type 2 diabetes mellitus during and after cancer treatment: A systematic review and meta-analysis

**DOI:** 10.1371/journal.pone.0176941

**Published:** 2017-05-03

**Authors:** Sophie Pettit, Elisabeth Cresta, Kirsty Winkley, Ed Purssell, Jo Armes

**Affiliations:** 1 College of Nursing, Midwifery and Healthcare, University of West London, London, United Kingdom; 2 Florence Nightingale Faculty of Nursing & Midwifery, King’s College London, London, United Kingdom; 3 Institute of Psychiatry, Psychology & Neuroscience, Kings College London, London, United Kingdom; Baylor College of Medicine, UNITED STATES

## Abstract

**Background:**

Cancer and Diabetes Mellitus (DM) are leading causes of death worldwide and the prevalence of both is escalating. People with co-morbid cancer and DM have increased morbidity and premature mortality compared with cancer patients with no DM. The reasons for this are likely to be multifaceted but will include the impact of hypo/hyperglycaemia and diabetes therapies on cancer treatment and disease progression. A useful step toward addressing this disparity in treatment outcomes is to establish the impact of cancer treatment on diabetes control.

**Aim:**

The aim of this review is to identify and analyse current evidence reporting glycaemic control (HbA1c) during and after cancer treatment.

**Methods:**

Systematic searches of published quantitative research relating to comorbid cancer and type 2 diabetes mellitus were conducted using databases, including Medline, Embase, PsychINFO, CINAHL and Web of Science (February 2017). Full text publications were eligible for inclusion if they: were quantitative, published in English language, investigated the effects of cancer treatment on glycaemic control, reported HbA1c (%/mmols/mol) and included adult populations with diabetes. Means, standard deviations and sample sizes were extracted from each paper; missing standard deviations were imputed. The completed datasets were analysed using a random effects model. A mixed-effects analysis was undertaken to calculate mean HbA1c (%/mmols/mol) change over three time periods compared to baseline.

**Results:**

The available literature exploring glycaemic control post-diagnosis was mixed. There was increased risk of poor glycaemic control during this time if studies of surgical treatment for gastric cancer are excluded, with significant differences between baseline and 12 months (p < 0.001) and baseline and 24 months (p = 0.002).

**Conclusion:**

We found some evidence to support the contention that glycaemic control during and/or after non-surgical cancer treatment is worsened, and the reasons are not well defined in individual studies. Future studies should consider the reasons why this is the case.

## Introduction

Incidence of diabetes mellitus (DM) continues to grow worldwide with 415 million estimated cases in 2015 and figures are predicted to reach 642 million by 2040 [1]. Current estimates in the United Kingdom suggest approximately 6% of the population has DM if both diagnosed and undiagnosed cases are included [2]. There is growing evidence that for individuals with DM, the risk of developing cancer significantly increases when compared to a non-diabetic population [3–5]. This is particularly the case for liver, pancreatic, colon/rectum, breast and bladder cancers [4]. For example, a threefold increase in the risk of developing colorectal cancer has been described in Type 2 DM (T2DM) populations [6].

Studies investigating consequences of cancer treatment on people with DM report worse outcomes when compared to non-diabetic counterparts. Consequences include: increased mortality [7,8], higher infection rates [9,10], higher hospitalisation rates [10], worse physical function [11] and poorer prognosis [12,9]. Potential reasons for these poorer outcomes include: prioritising cancer treatments over DM self-management activities [11,13]; increased prevalence of and/or under recognition of hyperglycaemia [14]; and clinicians lacking skills in managing both these complex conditions [14,15]. Sub-optimal DM management during cancer care can result in worsened glycaemic control [13]. This has been associated with lower overall survival [11,16] and may be explained, to some extent, by increased deaths from cardiovascular disease [17] and obesity.

Formal guidance on managing patients with comorbid cancer and DM is limited despite a number of epidemiological studies identifying an increased risk of developing cancer in people with T2DM [18,19] and potential interactions between treatments for these two diseases [20]. Likewise little is known about the short-term impact of cancer treatment/care on glycaemic control. One paper found that adherence to glucose lowering drugs decreased in patients following cancer diagnosis [21]. To improve outcomes for patients with both cancer and DM, health professionals need a greater understanding of the impact cancer treatment has on glycaemic control and concomitant DM self-management activities [11,13]. Consequently, this review was undertaken to answer the following question:

- Does glycaemic control worsen during treatment for cancer in people with T2DM?

## Methods

To answer the questions outlined above, we undertook a systematic review and meta-analysis of published quantitative studies investigating glycaemic control in patients with comorbid cancer and T2DM. We used HbA1c levels as an indication of glycaemic control. The recommended levels should be individually determined according to age, body weight, concomitant complications and diabetes duration. Most adults benefit from HbA1c ≤ 53 mmols/mol (7%) and a clinically relevant difference is considered to be change in HbA1c of 6mmols/mol, (0.5%) [22,23].

### Search strategy

We developed a broad search strategy (Table 1) to increase sensitivity. Adding further search terms resulted in key papers not being identified. The following databases were searched: Medline (1975 to February week 1, 2017), Embase (1975 to February week 1, 2017), PsychINFO (1975 to February week 1, 2017), CINAHL (1975 to February week 1, 2017), and Web of Science (1975 to February week 1, 2017). The search was limited to studies published after 1975, when glycated haemoglobin (HbA1c) plasma testing was introduced as a measure of glycaemic control. EC screened titles and abstracts of identified papers. Potentially eligible papers were reviewed independently by EC and JA.

**Table 1:** Search terms.

Type 2 Diabetes Mellitus		Cancer
OR		OR
• type 2 diabetes • glyc?emic control	AND	• Cancer • malignancy • neoplasm • melanoma • h?ematologic malignancy • lung cancer • GI disorder/digestive system • urinary tract cancer • gyn?ecologic cancer • breast cancer • colorectal cancer • prostate cancer • brain cancer • kidneys cancer • liver cancer
• HbA1c		
• blood glucose		
• hyperglyc?emia		
• Diabetes		

#### Eligibility criteria

Studies were included in the review if they:

- reported quantitative findings from randomised controlled trials, observational, cohort, longitudinal or cross sectional studies, and case note reviews
- were published in English language
- were full text studies
- investigated glycaemic control of T2DM during and up to five years after completing cancer treatment
- reported HbA1c levels
- included participants ≥ 18 years
- described blood glucose and DM complications

Studies were excluded in the review if they:

- included people with pancreatic cancer due to the higher proportion of pancreatic cancer patients having diabetes and glucose intolerance [24]
- investigated effects of cancer treatment on glycaemic control of Type 1 DM, where type 1 DM share a lesser number of concordant predictors with cancer then T2DM
- reported qualitative findings only

### Data extraction and quality appraisal

In accordance with the Preferred Reporting Items for Systematic Reviews and Meta-Analyses (PRISMA) guidelines [25], data on outcomes relating to glycaemic control in patients with comorbid cancer and T2DM were extracted systematically. We developed standardised forms, and study data were extracted by SP. Methodological study quality was assessed using the Effective Public Health Practice Project (EPHPP) Quality Assessment Tool for quantitative studies [26]. Studies were independently reviewed by two researchers (SP and JA) and disagreements were resolved through discussion. Studies were judged to be low quality if they had a small sample size, did not describe the study sample, did not describe the study time frame, did not justify the outcome measures used, failed to control for confounding variables or did not fully explain statistical analyses performed. Moderate to high-quality papers met some or all of these criteria. Low-quality papers were not excluded, but results are given less emphasis in the discussion. Tables summarising key findings were created.

### Meta-analysis

We were interested in measuring HbA1c (%/mmols/mol) change over time post baseline (diagnosis and/or cancer treatment initiation). The time points of interest were selected based on available data as follows; baseline (T0), one year post-baseline (T1), two years post-baseline (T2).

Of the papers eligible for inclusion the following data were extracted: mean, standard deviation (SD) and number of participants. Missing SDs were imputed using metagear etd software package in R. This uses the coefficient of variation from all complete cases to calculate missing values [27,28]. Datasets with imputed values were then analysed using a random effects model within the metafor software package [29]. This model does not make an inference based on just the studies analysed, but sees them as a random sample of studies from a larger population of studies that could or have been done; thus it allows for estimation of heterogeneity and inference to a broader population of studies.

To test if there was a statistically significant difference between the three time periods the means, SDs and variance estimates were extracted from each of the analyses; and a mixed-effects model analysis undertaken in metafor using the time period (baseline, one year following, two years following) as the moderator variable. The three pooled estimates were themselves entered into a fixed-effects regression model with the time point as the moderating factor. This allowed for calculation of both change over these time periods compared to the baseline, and the amount of heterogeneity explained by the model; as well as confidence intervals and p-values. Full data and code are shown in S1 Appendix.

## Results

**Fig 1:**
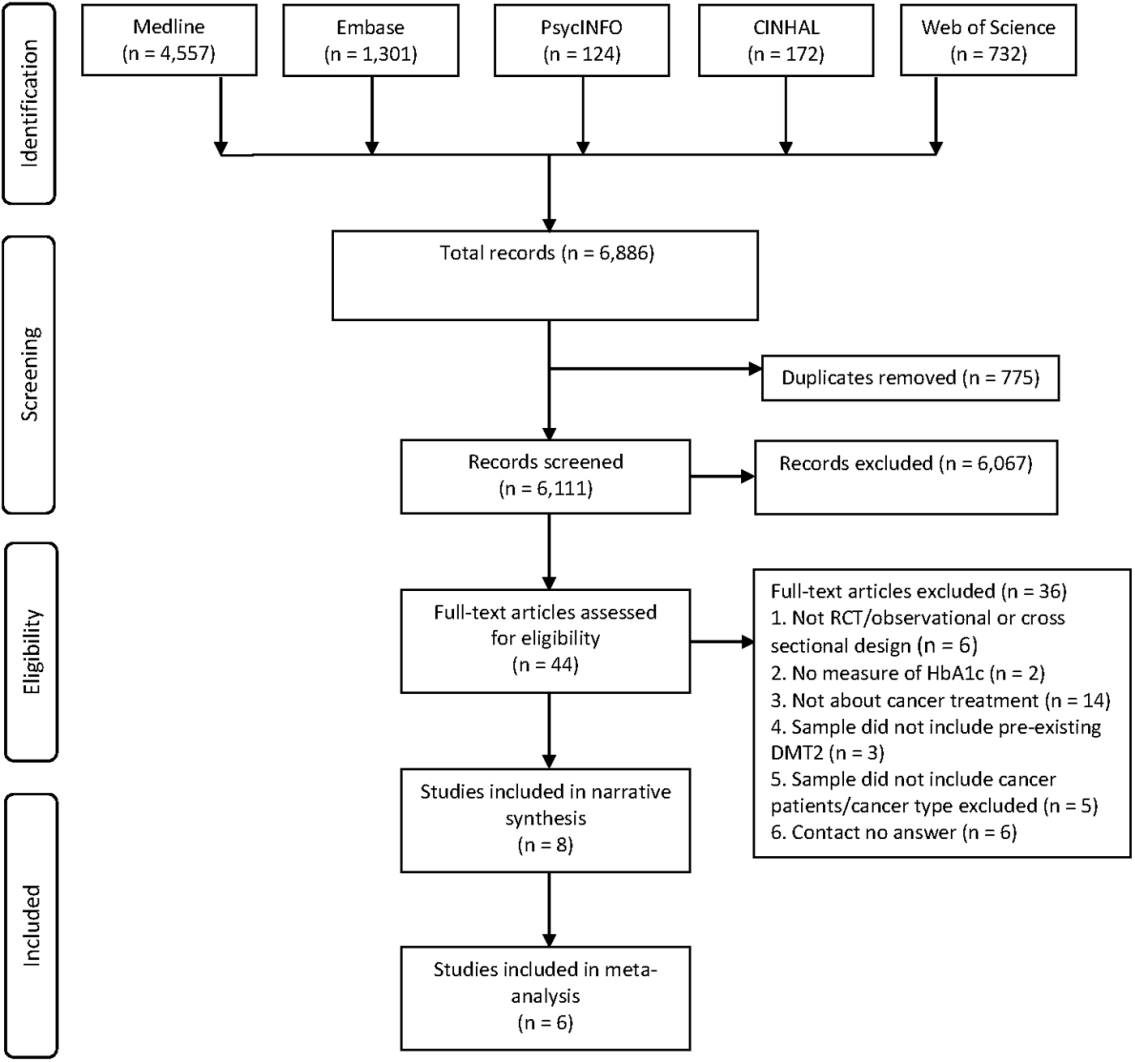
Data retrieval and assessment.

The literature search generated 6,886 potential papers of which 775 were duplicates. Following title and abstract review, 6,067 were excluded (Fig 1). Of 44 papers read in full, 36 were excluded and eight papers were selected for review (for further details on excluded papers, please refer to Fig 1).

### Study characteristics

Details of the eight included studies are provided in Table 2. Five were undertaken in the United States of America [30–34], one in South Korea [35], one in Taiwan [36] and one in China [37].

**Table 2:** Details of included studies.

Study (author, date, location)	Aim	Design/ Sample / time interval between first–last measure	Participant details	Details of cancer/ treatment	Demographics	Design	Measures	Analysis	Findings
An *et al*. 2013, South Korea	To evaluate the effect of gastrectomy on diabetes control in patients with T2DM	Retrospective review, Purposive 12 months	N = 64, Subtotal gastrectomy with gastroduode-nostomy	Early gastric cancer, Surgery (subtotal or total gastrectomy)	Mean age 62.7 years, Male, n = 43 (67.2%), Female, n = 21 (32.8%)	Retrospective review (cohort, one group pre and post)	*HbA1c, *Serum glucose, *Serum Insulin, *Fasting blood glucose, *HOMA-IR	*Categorical chi-square or Fisher exact test, *Continuous Mann-Whitney U test, *Kruskal-Wallis test, *Paired t-test, *Binary logistic regression	FBG levels 12 months post-gastrectomy were significantly lower than pre-operation levels (p = 0.001). No difference between HbA1c levels pre- and post-surgery. Homeostasis model assessment-estimated insulin resistance were significantly lower 12 months post-surgery than pre-surgery. Approximately 3.1% stopped diabetes medication and had HbA1c < 6.0% and FBS < 126 mg/dL. 54.7% decreased their medication and had reduced HbA1c or FBS.
Bayliss *et al*. 2011, USA	To assess the effect of breast, colon or prostate cancer on management of T2DM.	Retrospective review, Purposive 84 months	N = 582	Breast, Colon or Prostate cancer, stage 0, 1, 2 or 3, Not specified	Mean age 65.5 years, Male, n = 264 (45.4%), Female, n = 318 (54.6%)	Retrospective review (cohort, one group pre and post)	*HbA1c	*Mixed-effects models, *Multivariate linear regression, *Adjusted latent class analysis	Mean level of HbA1c did not change significantly from pre-diagnosis to post-diagnosis.
Calip *et al*. 2015, USA	To evaluate changes in oral DM medication adherence and glycaemic control for the year prior to breast cancer diagnosis (Year -1), during treatment, and in subsequent years.	Retrospective review, Purposive, 28 months	N = 509	Early stage (I and II) invasive breast cancer, Surgical procedure, chemo-therapy and endocrine therapy	Mean age 65.0 years, Female, n = 509 (100%)	Retrospective review (cohort, one group pre and post)	*HbA1c, *Adherence to DM medications, *Discontinuation rates	*T-test, *Fisher’s exact test, *Paired t-test, *McNemar exact tests	Compared with adherent users, non-adherent users of oral DM medications were more likely to be diagnosed with stage II tumours (p = .149). Non-adherent oral DM medication users were also more likely to be using greater than 4 cardiovascular disease medications (p = .035). MPR was lower in treatment period compared with year -1 (p < .001). Those that did not experience a discontinued episode was greatest in Year -1 (p < .001). Mean HbA1c and proportion not at goal HbA1c were higher during the treatment period (p = .001, p < .001) in comparison with year -1. Among adherent oral DM medication users, mean HbA1c was greater during the treatment period than in year -1 (p < .001). The proportion of non-adherent users with high HbA1c at year -1 was greatest in year +3 (p < .001).
Chou *et al*. 2012, Taiwan	The impact of pre-existing T2DM on patients with MM was evaluated by comparing clinical features, treatments and adverse reactions related to glycaemic control and OS of patients with and without diabetes.	Retrospective review, Purposive Not specified	N = 310, No history of diabetes, n = 40, Pre-existing diabetes, n = 270	Myeloma, Chemo-therapy	Mean age 71.8 years, Male, n = 226 (73.9%), Female, n = 84 (27.1%)	Retrospective review (cohort, two groups pre and post)	*HbA1c, *Glucose, *Diabetes related complication-s, *Antidiabet-ic therapies	*Pearson’s chi-squared test, *Landmark Kaplan-Meier estimate, *Log-rank test Cox proportional hazards, *Regression analyses	Patients with pre-existing DM had significantly higher proportion of renal impairment (p = .004). Patients with pre-existing DM had significantly higher Serum beta-2 microglobulin (p = .019). Significantly higher levels of SCr (p = .027) and eGFR (p = .021) and a higher proportion of CKD stage 4 and 5 (0.005) in pre-existing diabetes patients. Those with pre-existing diabetes had a significantly lower OS (p = .037).
Derweesh *et al*. 2007, USA	To investigate the incidence of new-onset DM and of worsening glycaemic control in established DM after starting ADT for prostate cancer.	Retrospective review, Purposive, Unclear–median 60.1 months follow-up	N = 369, No diabetes, n = 283, Pre-existing diabetes, n = 77, Newly diagnosed, n = 36	Prostate, ADT (surgical or medical)	Median age 73.2 years, Male, n = 396 (100%)	Retrospective review (cohort, two groups pre and post)	*HbA1c, *Fasting blood glucose	*Kruskal-Wallis, *Univariate / multivariate regression	Of the total number of patients, 19.4% had pre-existing diabetes. At a median follow up of 60.1 months, new onset DM was measured in 11.3% of patients previously without history. Pre-existing DM group had a significant increase in mean FBG levels after ADT (p < .001), HbA1c remained unchanged (p = .29).
Haidar *et al*. 2007, USA	The present study monitored the effects of ADT in men with T2DM on glycaemic control and on biochemical cardiovascular risk markers.	Retrospective review, Purposive 24 months	N = 29	Prostate ADT	Mean age 75.0 years, Male, n = 29 (100%)	Retrospective review (cohort, one group pre and post)	*HbA1c, *Fasting blood glucose, *Insulin dose requirements	*Non parametric Friedman test	With ADT, glycaemic control worsened with a significant increase in fasting glucose (T2, p < .01. T3, p = 0.000) and HbA1c (T2, p < .01. T5, P = .000). Increase in daily requirement of insulin (T2, p < .01. T5, p = .000). All biochemical cardiovascular risk markers significantly deteriorated (C-Reactive Protein, T2, p < .05. T5, p = .004; Fibrinogen, T2, p < .01. T5, p = .000; PAI-1, T2, p < .01. T5, p = .000; t-PA, T2, p < .01. T5, p = .000; Cholesterol, T3, p < .01. T5, p = .000; Triglycerides, T2, p < .01. T4, p = .000).
Keating *et al*. 2014, USA	To assess the effect of ADT on diabetes control, as measured by HbA1c levels and the intensification of diabetes pharmacotherapy in men with prostate cancer.	Retrospective review, Purposive 24 months	N = 4474, DM, No ADT; N = 2237, DM, ADT N = 2237	Prostate ADT	Mean age non-ADT, 66.0 years, Mean age ADT, 70.2 years	Retrospective review (cohort, two groups pre and post)	*HbA1c, *Changes to diabetic medications	*Multiple imputation, *Propensity score analysis and logistic regression to match non-ADT/ADT pairs, *Paired student t-tests of difference in differences for HbA1c change, *Incidence rate of new medications for DM, *Cox proportional hazards regression model	HbA1c increased at 1 year for men treated with ADT and decreased for non-users for a difference in differences of +0.24 (p = 0.008). Results were similar at 2 years (+0.18; p = 0.03). Higher unadjusted rate for initiating or increasing a new drug class for men on ADT then non-users (p < 0.001). Initiation or addition of insulin higher among ADT then non-users (p = 0.05). ADT associated with increased hazard of the addition of diabetes medication (p < .001).
Liu *et al*. 2015, China	To investigate the effectiveness of radical gastrectomy with modified gastric by-pass surgery in treating gastric cancer patients with type 2 DM	Retrospective review, Purposive, 12 months	N = 93	Gastric, A–BMI >28kg/m^2^ (n = 30); B–Billroth I anastomosis (n = 21); C–Roux-en-Y anastomosis and BMI >28kg/m^2^ (n = 25); D–BMI <28kg/m^2^ (n = 17)	Mean age 59 years, Male, n = 46 (47.9%), Female, n = 50 (52.1%)	Retrospective review (cohort, one group pre and post)	*HbA1C, *FBG, *2 hour postprandial glucose, *BMI, *C-peptide	*T-*test	Decrease in FBG at 6 and 12 months post-surgery in groups A and D (p < 0.01) and at 12 months in group C (p < 0.05). Decrease in 2 hour postprandial glucose at 6 and 12 months post-surgery in groups A and D (p < 0.01) and group C (p < 0.05). Decrease in BMI at 6 and 12 months post-surgery in groups A and C (p < 0.05). Decrease in HbA1C at 6 and 12 months post-surgery in groups A and D (p < 0.01). Decrease in C-peptide at 6 and 12 months post-surgery in groups A, C and D (p < 0.01).

* ADT = Androgen Deprivation Therapy

* BMI = Body Mass Index

* DM = Diabetes Mellitus

* eGFR = estimated Glomerular Filtration Rate

* FBG = Fasting Blood Glucose

* HbA1c = glycated haemoglobin

* HOMA-IR = Homeostatic Model Assessment-estimated insulin resistance

* MPR = Medication Possession Ratio

* OS = Overall Survival

* SCr = Serum Creatinine

* T = Time

* T2DM = Type 2 Diabetes Mellitus

* PAI-1 = Plasminogen Activator-1

All eight studies employed a retrospective review of case notes and/or medical information design focused on a convenience sample of patients with cancer and pre-existing T2DM. Of these, five studies used a within group comparison using the same participants who had T2DM before and after receiving cancer treatment [30,31,33,35,37]. Three studies compared cancer patients with pre-existing T2DM to cancer patients with no history of T2DM, before and after cancer treatment [32,34,36]. A total of 6,433 people participated in the included studies and sample sizes were diverse, ranging from 29 [33] to 4,474 participants [34]. Participants’ mean age ranged from 59.0 years [37] to 75.0 years [33]. Based on diagnostic groups studied; three studies included male-only participants [32–34], one study included female-only participants [31] and four studies included both male and female participants [30,35,36,37].

All studies assessed the impact of cancer diagnosis and/or cancer treatment on T2DM outcome, however timing of assessments varied between studies. Whilst for two studies [32,36] time frames were unclear, for the remaining studies the time interval from first to last data collection points ranged from 12 [35,37] to 84 months [30]. Most studies focused on one cancer type, with the exception of one study which included patients with either breast, colon or prostate cancer [30]. Of those studies focusing on one cancer type, three included men with prostate cancer [32–34], one included people with myeloma [36], one included women with breast cancer [31] and two studies included people with gastric cancer [35,37].

#### Measurement of glycaemic control

All selected studies routinely assessed HbA1c (%/mmols/mol) as a long term determinant of glycaemic control. Mean percentage HbA1c level (and mmol/mol) at different time points are described in Table 3 and Table 4.

**Table 3:** Mean percentage (and mmol/mol) HbA1c across study time points.

	Time point								
Author, Year (Sample size)	Diagnosis *Treatment*	Pre-base-line	Baseline* (Diagnosis and/or treatment start)	Post-baseline (in months)					
				3	6	12*	24*	36	60
An et al. 2013	Early gastric cancer, *Surgery*								
STG B I^a^ (n = 36)			7.2 *(55)*	6.8 *(51)*	7.0 *(53)*	7.1 *(54)*			
STG B II^b^ (n = 16)			7.3 *(56)*	6.9 *(52)*	7.1 *(54)*	7.1 *(54)*			
Total G^c^ (n = 12)			7.1 *(54)*	6.5 *(48)*	6.5 *(48)*	6.5 *(48)*			
Calip et al. 2015 (n = 399)	Stage I and II breast cancer, *Surgery*, *chemo-therapy*, *endocrine therapy*	7.0 *(53)*	7.3 *(56)*			7.4 *(57)*	7.4 *(57)*	7.3 *(56)*	
Chou et al. 2012 (n = 34)	Myeloma *Chemotherapy*		7.1 *(54)*						
Derweesh et al. 2007 (n = 77)	Prostate ADT* *(surgical or medical)*		7.1 *(54)*						7.2 *(55)*
Haidar et al. 2007 (n = 29)	Prostate *ADT*		6.3 *(45)*						
Keating et al. 2014 (n = 2,105)	Prostate *ADT*		7.2 *(55)*			7.4 *(57)*	7.4 *(57)*		
Liu et al. 2015	Gastric cancer, *Surgery*								
A (n = 30)			9.5 (1.0)		5.4 (0.6)	4.6 (0.4)			
B (n = 21)			9.1 (1.1)		8.8 (0.7)	9.2 (1.2)			
C (n = 25)			8.9 (0.9)		7.1 (0.8)	7.8 (0.5)			
D (n = 17)			9.6 (1.0)		5.8 (0.5)	4.8 (0.3)			

* Time points selected for comparison in meta-analysis

^a^ Subtotal gastrectomy with gastroduodenostomy

^b^ Subtotal gastrectomy with gastrojejunostomy

^c^ Total gastrectomy

* Androgen Deprivation Therapy

**Table 4:** Median percent (%) HbA1c across study time points.

Time points	-24 pre diagnosis to -6 months	-6 months to diagnosis (0)	Diagnosis (0) to 6 months	6 months to 12 months	12 months to 24 months	24 months to 60 months
Bayliss et al. 2011 (n = 553)	7.9 *(63)*	7.6 *(60)*	7.7 *(61)*	7.8 *(62)*	7.9 *(63)*	7.8 *(62)*

In the study by Haidar and colleages [33], between five and eight HbA1c levels were recorded throughout a 24 month period (mean HbA1c = 9.3%, *78 mmol/mol*).

Four studies also recorded fasting blood glucose (FBG) [32,33,35,37], two studies recorded serum glucose [35,36], one study recorded serum insulin and homeostasis model assessment-estimated insulin resistance (HOMA-IR) [35]; one study recorded administered insulin dose [33]; and one study recorded BMI and C-peptide levels [37]. Adherence and changes to diabetes medication were reported in three studies [31,34], as well as information on diabetes-related complications assessed by occurrence of neuropathy, nephropathy and retinopathy [36].

### Quality assessment

Most studies were either of moderate [30,31,35] or weak quality [32,33,36,37] and one was judged to be of strong quality [34]. Detailed evaluation of each study using the EPHPP criteria is shown in Table 5.

**Table 5:** Quality assessment of included studies.

Study	Selection bias	Study design	Confounds	Data collection methods	Withdrawals and drop outs	Statistical analyses suitable	Global rating
An *et al* (2013)	Moderate	Weak	Strong	Moderate	Strong	Yes	Moderate
Bayliss *et al*. (201)	Moderate	Moderate	Strong	Moderate	Weak	Yes	Moderate
Calip *et al*. 2015	Moderate	Moderate	Weak	Moderate	Moderate	Yes	Moderate
Chou *et al*. 2012	Weak	Moderate	Weak	Strong	Weak	Yes	Weak
Derweesh *et al*. 2007	Weak	Weak	Weak	Moderate	Weak	Yes	Weak
Haidar *et al*. 2007	Weak	Weak	Weak	Weak	Weak	Yes	Weak
Keating *et al*. 2014	Moderate	Moderate	Strong	Strong	Moderate	Yes	Strong
Liu *et al*. 2015	Weak	Moderate	Weak	Strong	Weak	Yes	Weak

Generally, papers described as being of weak quality had small sample sizes from a single clinical setting [33], failed to describe if data were missing and/or how this was dealt with [32,33,36,37], excluded cases lacking complete data without sufficiently describing details of the process [33] and did not appropriately describe or deal with confounding variables [32,33,36,37]. Papers described as being of moderate quality failed to provide sufficient detail on the patients’ cancer such as type and stage [30,35], details of how patients were selected and why [35], how missing data was dealt with [30], or did not appropriately deal with confounding variables [31].

#### Glycaemic control

Four studies found no significant difference in HbA1c levels for diabetic patients before and after cancer treatment initiation [30,35] or diabetic patients before and after cancer treatment initiation compared to non-diabetic controls [32,36]. Two studies reported an increase in HbA1c after androgen deprivation therapy (ADT) was initiated [33,34] and one study described raised HbA1c levels following diagnosis and treatment (not specified) for breast cancer [31]. One study in gastric cancer showed mixed results according to type of surgical procedure and/or BMI, however the study design was judged to be weak [37].

Of the four studies measuring FBG, two described a significant increases in FBG at 24 months [33] and 60 months [32] after receiving a course of ADT. However both were evaluated as weak quality studies and reported results for time bands rather than specified time points. For people with gastric cancer one study reported a significant decrease in FBG within 12 months following gastrectomy [35] whilst the other showed reduction only for those with a high BMI who underwent a total gastrectomy and modified Roux-en-y anastomosis and those with normal BMI who underwent a total gastrectomy and standard Roux-en-y anastomosis [37].

In three studies measuring insulin resistance, one study described a significantly lower resistance to insulin post-gastrectomy [35] and two studies reported increased insulin requirements after patients commenced ADT [33,34].

#### Risks and side effects

Adherence to diabetic medications from the time of cancer diagnosis was described in three studies. For current DM medication users, patients reported they either ceased taking DM medications [35], or reduced DM medications use after their cancer diagnosis [31,35]. The proportion of patients achieving recommended HbA1c levels decreased following worsened adherence to DM medication after being diagnosed with breast cancer [31]. Following diagnosis, one study reported that prostate cancer patients were prescribed new DM medication, or changed their current DM medication to a new class once commencing ADT [34]. Drug classes included metformin, sulfonylureas, other oral drugs and insulins.

Women with breast cancer identified as being non-adherent with oral T2DM medication were more likely to be taking ≥4 cardiovascular disease medications then adherent users [31].

### Meta-analysis

Six of the seven papers included in the systematic review were included in the meta-analysis. The paper by Haidar and colleagues [33] was excluded as it only reported median and range, the data was too heterogeneous, the study time points did not match those of interest and no response was received from the corresponding author in attempt to clarify these points. The paper by Bayliss and colleagues [30] was excluded as the data were too heterogeneous and the study time points did not match those of interest.

We ran three meta-analyses for time points at which data across studies could be clustered together for comparison (see Table 3). Time 0 (T0) (baseline from diagnosis/treatment initiation) included data from six studies [31,32,34,35,36,37]. Time 1 (T1) (12 months post baseline) included data from four studies [31,34,35,37]. And time 2 (T2) (24 months post baseline) included data from two studies [31,34].

We compared the mean % *(mmol/mol)* HbA1c levels and SDs. Although all studies provided mean scores, SDs were missing from some and so we calculated SDs from standard errors where provided [31,34]. For studies which provided means and ranges [32,35,36] SDs were imputed using metagear [38].

**Fig 2:**
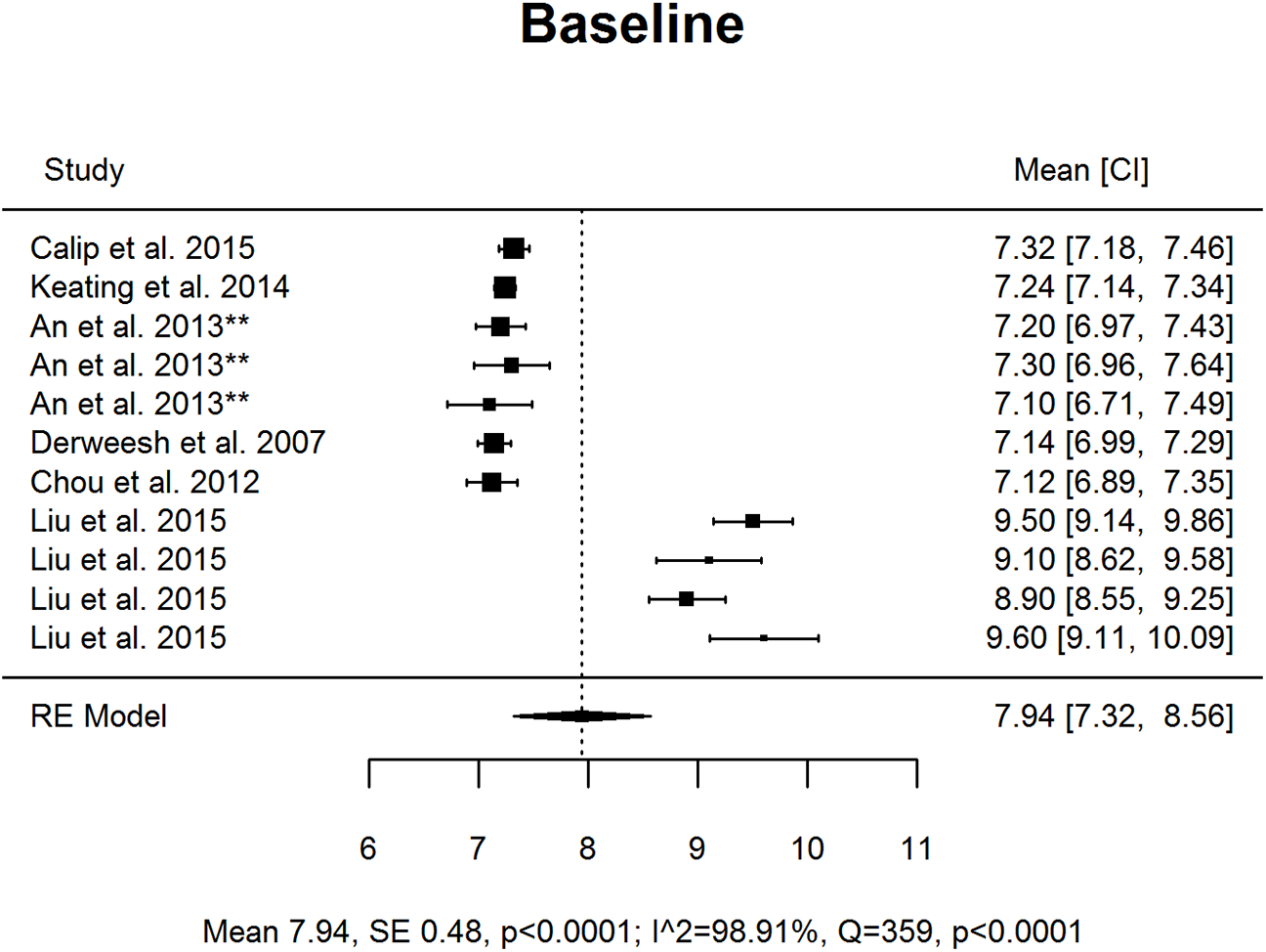
Forest plot of studies considering mean HbA1c levels (%) at time of cancer diagnosis and treatment initiation.

**Fig 3:**
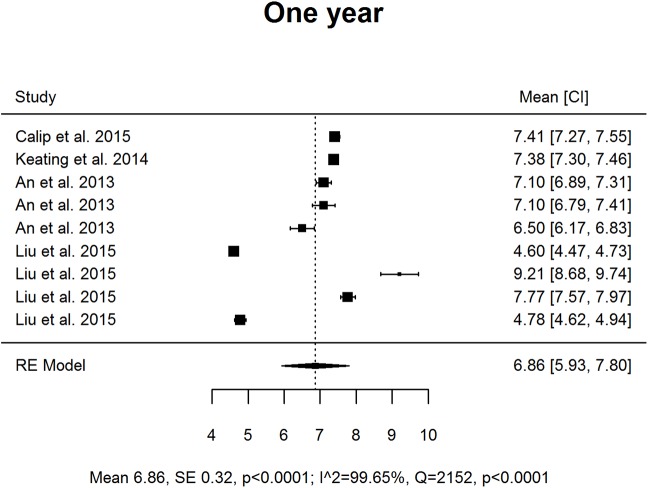
Forest plot of studies considering mean HbA1c levels (%) at 12 months post time of cancer diagnosis and treatment initiation.

**Fig 4:**
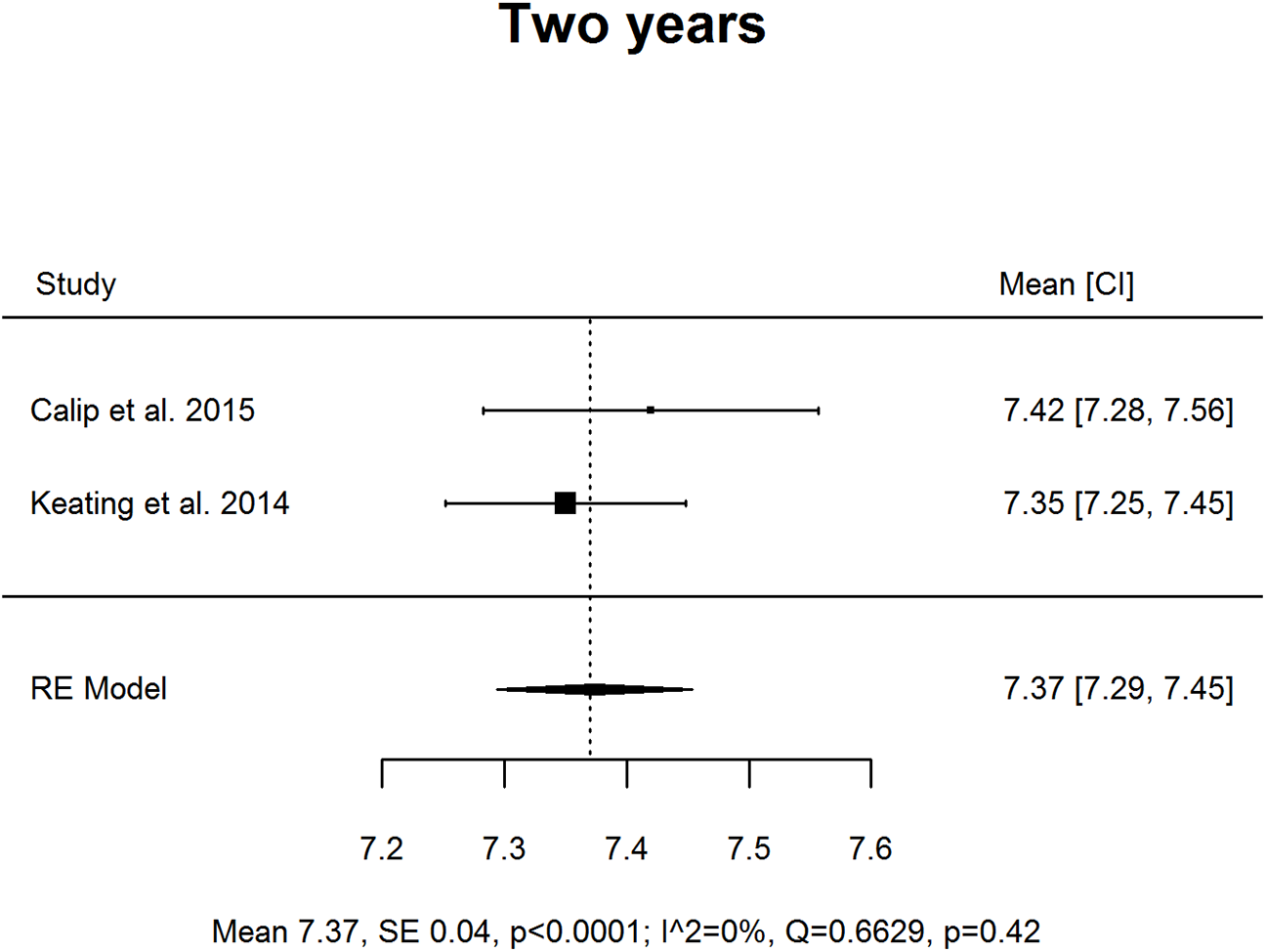
Forest plot of studies considering mean HbA1c levels (%) at 24 months post time of cancer diagnosis and treatment initiation.

**Fig 5:**
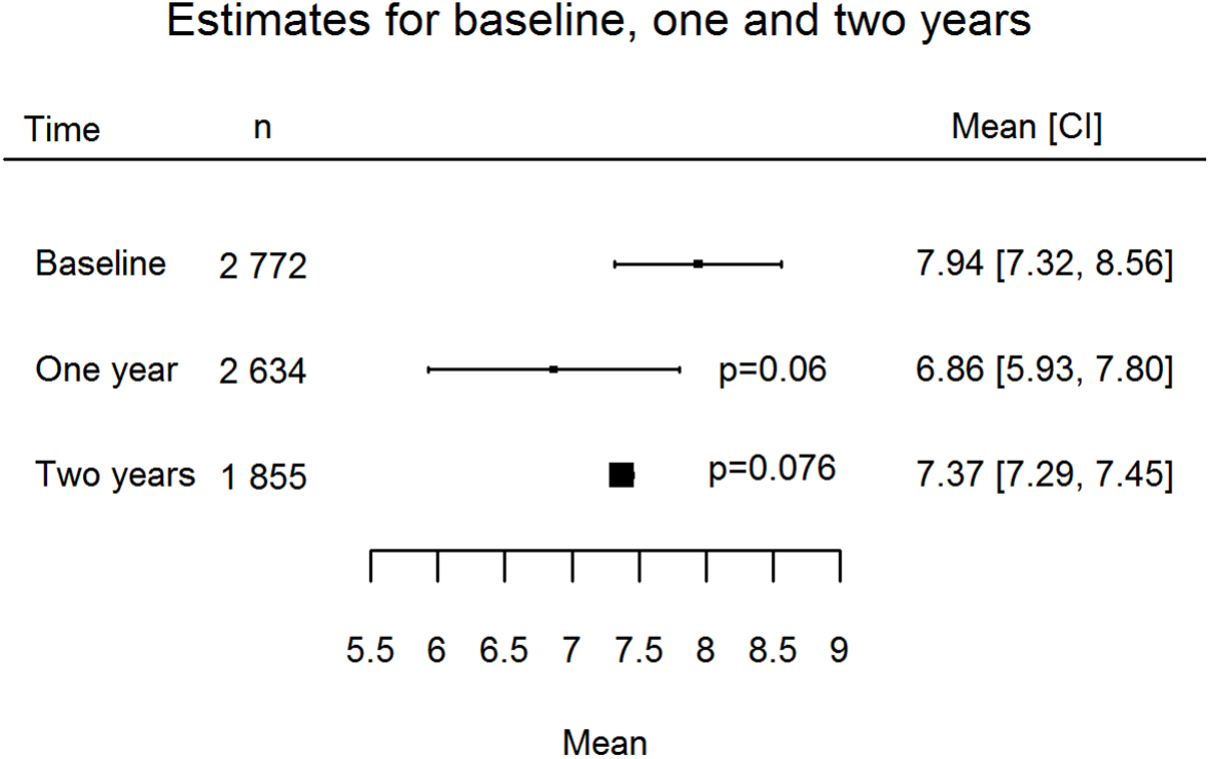
Forest plot of collapsed studies considering HbA1c levels at baseline and two-year estimates.

Results are presented as forest plots (Figs 2–5). At baseline mean HbA1c was 7.94% (63 *mmol/mol;* 95% CI 7.32, 8.56) and subject to high levels of heterogeneity (Q = 359.11 df = 10, p < .0001, I^2^ 98.91%). At T1 mean HbA1c was 6.86% (*52 mmol/mol;* 95% CI 5.93, 7.80), and subject to high levels of heterogeneity (Q = 2151.52, df = 8, p < .0001, I^2^ 99.65%). At T2 the mean was 7.37% (57 *mmol/mol;* 95% CI 7.29, 7.45), this was less heterogeneous but only included two studies (Q = 0.66, df = 1, p = 0.42, I^2^ 0%).

The analysis was repeated excluding the study by An and colleagues [35] and Liu and colleagues [37] because of the effects of gastric cancer surgery on weight loss. At baseline the mean HbA1c was 7.21% (*55 mmol/mol;* 95% CI 7.13, 7.29) and subject to moderate levels of heterogeneity (Q = 5.20 df = 3, p = 0.16, I^2^ 41.38%). At T1 the mean HbA1c was 7.39% (*57 mmol/mol;* 95% CI 7.32, 7.46) and subject to low levels of heterogeneity but only included two studies (Q = 0.14, df = 1, p = <0.71, I^2^ 0%). At T2 the mean HbA1c was 7.37% (*56 mmol/mol;* 95% CI 7.29, 7.45), again with low levels of heterogeneity but only including two studies (Q = 0.66, df = 1, p = 0.42, I^2^ 0%). There was a significant difference between T0 and T1 estimates (p < 0.001) and T0 and T2 estimates (p = 0.002).

## Discussion

Results from the meta-analysis demonstrate deterioration in HbA1c levels at 24 months post-cancer diagnosis and treatment initiation. When limiting the analysis to exclude studies with populations receiving gastric surgery, HbA1c levels increase at both 12 and 24 months in comparison to baseline.

All studies included routinely assessed HbA1c as a long term determinant of blood glucose levels. Four studies found no significant difference in HbA1c levels before and after cancer treatment [30,32,35,36], three reported an increase in HbA1c levels following cancer diagnosis and treatment [31,33,34] and one reported mixed results depending on participants BMI and type of surgical procedure undergone [37]. This discrepancy may be explained by differences in BMI resulting from the specific cancer diagnosis and/or its corresponding treatment. For example, the first line treatment for gastric cancer is radical surgery which removes some or all of the stomach and limits food intake [35,37]. An alternative explanation for this discrepancy might be that clinically significant changes in glycaemic control necessitated adjustment of diabetes therapy however this was not reflected in changes in HbA1C.

Differences in cancer type and cancer treatment across studies may also explain inconsistencies in the results between the studies. For example, the three studies which highlighted adverse effects on glycaemic control included populations that may have received hormone therapy as a part of their cancer care [31,33,34]. Two studies described a significant increase in FBG at up to 24 [33] and 60 months [32] after receiving a course of ADT, suggesting an increase in insulin resistance. On the other hand, two studies [35,37] reported a significant decrease in FBG within 12 months following gastrectomy further suggesting that cancer type and corresponding treatment will have differing effects on glycaemic control. Chemotherapy drug regimens vary according to cancer type/stage however none of the studies provided information on this. Thus it is possible that differences in glycaemic control arise from different treatment regimens being compared. Likewise many patients are administered steroids as part of an anti-emetic regimen however due to the lack of information provided by the included studies it is not possible to assess the impact of this on glycaemic control. Further research is required to investigate this in more detail.

Considerable differences between studies may explain the lack of consistency in the association between HbA1c levels and initiation of cancer treatment. For example, the timing of HbA1c recordings relative to the timing of their cancer diagnosis varied greatly across the studies. The study by Chou and colleagues [36], which was rated as being of weak quality, only measured HbA1c at baseline, therefore there was no comparison. Whilst Derweesh and colleagues [32], also judged as being of weak quality, assessed HbA1c on more than one occasion (baseline and 60 months post-diagnosis or treatment initiation), it is possible patients had either fully recovered by the second assessment or had sufficient time to adapt to these two concurrent illnesses. Generally there was variation between studies in relation to: cancer diagnosis (type, stage, severity), cancer treatment (chemotherapy, endocrine therapy, radiotherapy, surgery, ADT), competing comorbidities (obesity and cardiovascular disease), attention to potential confounders (duration of DM, treatment history, supportive medications) and inconsistencies in inclusion and exclusion criteria, dealing with missing data and use of a control group for comparison.

Poor outcomes such as hyperglycaemia have been reported in patients with comorbid cancer and diabetes [14] but it should be noted that there is little available data to date. For patients with comorbid cancer and diabetes, the adverse outcomes highlighted in this review include higher mortality rates [36], consistent with findings in several studies [7,8,11,16] and poor adherence to DM medications [31,34,35] as reported by Hershey and colleagues [11].

Poorer adherence to diabetic medications post-cancer diagnosis was described in three studies and patients reported they either ceased taking DM medications [35], or reduced DM medications use after diagnosis [31,35]; and this supports findings from the study by Zanders and colleagues [21]. An explanation for this may be that little attention is paid to glycaemic control by cancer health professionals and/or poorer self-management by the patients themselves when also burdened with the added responsibilities and strains associated with other competing chronic conditions [30] including cancer self-management [13], however evidence to support this is extremely limited. It is also possible that there is a lack of integrated care and competing care priorities but again, evidence to support this is limited. It would be useful to know how this group of people assess and decide between competing care priorities. Being able to differentiate between modifiable and non-modifiable factors at both the cancer and T2DM level may help health professionals to identify how best to support and intervene with this group of people.

### Limitations and recommendations

The findings of this meta-analysis are limited by the small number of studies reporting on the impact of cancer treatment on glycaemic control and adverse outcome in people with T2DM. This is further compounded by methodological issues and inconsistencies identified in the seven included studies. All used a retrospective design incorporating routinely collected data which varied in terms of data completeness and also time points at which data collection occurred relative to cancer treatment. As the studies were observational there was no suitable comparison group and it is possible that glycaemic control may worsen in participants’ without a cancer diagnosis over the 12 to 24 month period [39]. Generally it is not clear whether studies included all patients who passed through the system, or excluded those with missing information. Inconsistencies in key clinical variables made comparisons between studies difficult. A prospective study design may have better resolved some of these issues. Finally we initially intended to evaluate whether DM complications are greater in people with T2DM who receive cancer treatment, however this was not possible due to the limited evidence available.

### Concluding remarks and implications for research

This meta-analysis found that following treatment for cancer (and particularly ADT) there is a small statistically significant increase in HbA1c in people with pre-existing T2D when gastric surgery cases are removed. Whilst this increase was not clinically meaningful, results should be treated with caution due the lack of high quality evidence available for review. The limited research conducted evaluating glycaemic control in cancer patients with T2DM means it is not possible to judge potentially serious outcomes as a result of treatment interactions in this population. Understanding such potential interactions and outcomes could help inform decisions made by health care professionals regarding treatments and care pathways. Future research is required to investigate glycaemic control during cancer treatment and what happens to HbA1c levels and DM complications during steroids and cytotoxic and antiemetic regimes. The research may help inform best care for patients with comorbid cancer and DM.

## Supporting information

S1 Appendix Full data and code (metafor). (PDF)

S1 Table PRISMA check list. (PDF)
