# Supplementary material for: Glycaemic control in people with type 2 diabetes mellitus during and after cancer treatment: A systematic review and meta-analysis
Source: PLoS One. 2017 May 3;12(5):e0176941. doi: 10.1371/journal.pone.0176941 (PMC5415164; doi:10.1371/journal.pone.0176941)
Supplement: S1 Appendix — (PDF) [file pone.0176941.s001.pdf]

S1 Appendix. Full data and code (metafor)

```
> library(meta)
> library(metafor)
> mab<-rma(mi=MEAN, sdi=SD, ni=NUMBER, measure="MN", data=base)
> maT1<-rma(mi=MEAN, sdi=SD, ni=NUMBER, measure="MN", data=T1)
> maT2<-rma(mi=MEAN, sdi=SD, ni=NUMBER, measure="MN", data=T2)
>
>
##################################################################################
> ####plots###
> tiff(file="Base.tiff", res=300, height=1200, width=1500)
> forest(mab, cex=0.7, main="Baseline", slab=base$NAME,
+ refline=7.94, xlab="Mean 7.94, SE 0.48, p<0.0001; I^2=98.91%, Q=359, p<0.0001")
> text(3.4, 13, "Study", cex=0.7)
> text(12.5, 13, "Mean [CI]", cex=0.7)
> dev.off()
null device
          1
>
> tiff(file="T1.tiff", res=300, height=1200, width=1500)
> forest(maT1, cex=0.7, main="One year", slab=T1$NAME,
+ refline=6.86, xlab="Mean 6.86, SE 0.32, p<0.0001; I^2=99.65%, Q=2152, p<0.0001")
> text(-0.9, 10.7, "Study", cex=0.7)
> text(13.5, 10.7, "Mean [CI]", cex=0.7)
> dev.off()
null device
          1
>
> tiff(file="T2.tiff", res=300, height=1200, width=1500)
> forest(maT2, cex=0.7, main="Two years", slab=T2$NAME,
+ refline=7.37, xlab="Mean 7.37, SE 0.04, p<0.0001; I^2=0%, Q=0.6629, p=0.42")
> text(6.95, 3.35, "Study", cex=0.7)
> text(7.76, 3.35, "Mean [CI]", cex=0.7)
> dev.off()
null device
          1
> #########################################
> ####combined plot###
> names<-c("Baseline", "One year", "Two years")
> yia<-c(7.9425, 6.8640, 7.3737)
> sea<-c(0.3175, 0.4781, 0.04070)
> n<-c("2 772", "2 634", "1 855")
>
> tiff(file="TB12.tiff", res=300, width=1300, height=1100)
> forest(x=yia, sei=sea, cex=0.7, slab=names, xlab="Mean",
+ ilab=n, ilab.xpos=4.7)
> text(8.5, 1.99, "p=0.06", cex=0.7)
> text(8.5, 1.05, "p=0.076", cex=0.7)
> text(3.2, 4.3, "Time", cex=0.7)
> text(10.4, 4.3, "Mean [CI]", cex=0.7)
> text(4.7, 4.3, "n", cex=0.7)
> text(7.2, 5.3, "Estimates for baseline, one and two years",
+ cex=0.9)
> dev.off()
null device
          1
>
> dat.comp <- data.frame(estimate = c(7.9425, 6.8640, 7.3737),
+ stderror = c(0.3175, 0.4781, 0.04070),
+ time = c("base","one", "two"), tau2 = c(1.0823 , 2.0388, 0))
> dat.comp
  estimate stderror time   tau2
1   7.9425   0.3175 base 1.0823
2   6.8640   0.4781  one 2.0388
```

```
3   7.3737   0.0407  two 0.0000
> mod1<-rma(yi=estimate, sei=stderror, mods = ~ time, method="FE",
+ data=dat.comp, digits=3)
> mab

Random-Effects Model (k = 11; tau^2 estimator: REML)

tau^2 (estimated amount of total heterogeneity): 1.0823 (SE = 0.4960)
tau (square root of estimated tau^2 value):      1.0404
I^2 (total heterogeneity / total variability):   98.91%
H^2 (total variability / sampling variability):  91.79

Test for Heterogeneity:
Q(df = 10) = 359.1122, p-val < .0001

Model Results:

estimate       se     zval     pval    ci.lb    ci.ub
  7.9425   0.3175  25.0125   <.0001   7.3202   8.5649      ***

---
Signif. codes:  0 ‘***’ 0.001 ‘**’ 0.01 ‘*’ 0.05 ‘.’ 0.1 ‘ ’ 1

> maT1

Random-Effects Model (k = 9; tau^2 estimator: REML)

tau^2 (estimated amount of total heterogeneity): 2.0388 (SE = 1.0284)
tau (square root of estimated tau^2 value):      1.4279
I^2 (total heterogeneity / total variability):   99.65%
H^2 (total variability / sampling variability):  286.61

Test for Heterogeneity:
Q(df = 8) = 2151.5158, p-val < .0001

Model Results:

estimate       se     zval     pval    ci.lb    ci.ub
  6.8640   0.4781  14.3577   <.0001   5.9270   7.8010      ***

---
Signif. codes:  0 ‘***’ 0.001 ‘**’ 0.01 ‘*’ 0.05 ‘.’ 0.1 ‘ ’ 1

> maT2

Random-Effects Model (k = 2; tau^2 estimator: REML)

tau^2 (estimated amount of total heterogeneity): 0 (SE = 0.0052)
tau (square root of estimated tau^2 value):      0
I^2 (total heterogeneity / total variability):   0.00%
H^2 (total variability / sampling variability):  1.00

Test for Heterogeneity:
Q(df = 1) = 0.6629, p-val = 0.4155

Model Results:

estimate       se     zval     pval    ci.lb    ci.ub
  7.3737   0.0407 181.2417   <.0001   7.2940   7.4534      ***

---
Signif. codes:  0 ‘***’ 0.001 ‘**’ 0.01 ‘*’ 0.05 ‘.’ 0.1 ‘ ’ 1

> mod1

Fixed-Effects with Moderators Model (k = 3)

Test for Residual Heterogeneity:
```

```
QE(df = 0) = 0.000, p-val = 1.000

Test of Moderators (coefficient(s) 2,3):
QM(df = 2) = 4.327, p-val = 0.115

Model Results:

         estimate     se    zval   pval   ci.lb  ci.ub
intrcpt     7.942  0.317  25.016  <.001   7.320  8.565  ***
timeone    -1.078  0.574  -1.879  0.060  -2.203  0.046    .
timetwo    -0.569  0.320  -1.777  0.076  -1.196  0.059    .

---
Signif. codes:  0 ‘***’ 0.001 ‘**’ 0.01 ‘*’ 0.05 ‘.’ 0.1 ‘ ’ 1

>
```
